# A Letter to John Bull on the Dissection of His Body

**Published:** 1829-10-01

**Authors:** 


A Letter to John Bull on the Dissection of his Body. By Gracchus.

We are daily more and more convinced that the rejection of Mr. Warburton's bill will be the destruction of all rational hopes of a better one in the ensuing session. Its suspension has excited the passions and the clamours of the senseless multitude, the designing knaves, the enemies of medical science, and, perhaps worse than all, the speculations and propositions of fools. The letter before us is a pretty specimen! The writer assumes that the only methods which either the advocates or opponents of anatomy can propose, are the following:—

"1st. Forbidding dissection altogether, and sending all students to foreign lands for instruction. 2. Giving up the bodies of suicides and all persons executed or otherwise dying whilst suffering the punishments of the law. 3. Making use of the bodies of surgeons, either with or without those of the preceding class. 4. Giving up the bodies for dissection of those who die unclaimed in the workhouses. 5. Legalizing the sale of dead bodies. 6. Legalizing exhumation. 7. Passing my bill."

Of the 7 propositions, the 3d is the only feasible one—though, as things now stand, there is some danger of the *first* proposition being verified! We doubt much whether Gracchus be in his right senses—for really many of his arguments smack of Bedlam. Thus he declaims in good set terms against the plan of giving up unclaimed bodies for dissection, averring that "this alternative has something very horrible on the face of it to any man who feels and reverences the *spirit* of the English constitution"!—He deprecates the said plan as "a stigma on poverty," and talks about the feelings of the dying and friendless poor, with wonderful sympathy! Poor man. He evidently has never been at the bed-side of the dying, or he would know that not one in fifty has the least idea of death up to the very last moment of his existence.

But let us see the remedy which Gracchus proposes—in other words, read "my bill."

"Whereas, it is expedient for the better preservation of the health of the people of these realms, that medicine and surgery be encouraged; and whereas, it is established, by the testimony of the learned of all nations, that these sciences can only be acquired or improved by the constant dissection of dead human bodies, Be it enacted,

"'1. That it shall not be lawful to inter in any vault, church-yard, or other place of public sepulture, the body of any man or male child, dying in the United Kingdom, until the said body shall have been opened by a physician, surgeon, or apothecary.

"'2. That this examination shall be made in the premises of the said physician, surgeon, or apothecary, or in any other place perfectly separate and distinct from the house the deceased died in, as may be agreed on betwixt the said physician and the friends of the deceased.

"'3. That the relation of the deceased, or if he have no relations, any person wishing to bury him, or if no such person appear within two days after his death, the overseers of the parish wherein he died, shall have the following powers:—1. They shall dictate how many and what persons, besides the physician, surgeon, or apothecary, who opens the body, shall be present during such examination. 2. They shall fix (whether they are present or by their deputies) peremptorily the limit beyond which incisions into the body of the deceased shall not be carried. 3. They shall appoint the day, and the time of the day, at which the examination shall be made; always providing that longer than one fortnight shall not elapse between a man's death and the aforesaid examination of his body.

"'4. That it shall be imperative on every surgeon to perform the examination of a dead body, or cause the same to be performed by some other surgeon, within two days after receiving a written notice.

"'5. That every physician, surgeon, or apothecary, that opens a body, shall endeavour to ascertain the cause of the deceased's death, and shall transmit to the minister of the parish, wherein the deceased is to be buried, a certificate of the form mentioned below touching this matter, and that he shall keep a copy of such certificate by him, for transmission when required, either to his own college, or such other place, or officer, as his Majesty's Privy Council may direct.

"'6. That the office of searchers be abolished.

"'7. Provided always that nothing herein stated shall be understood to forbid the opening of the bodies of women and female children, at the habitations in which they die, or at any other places whereunto their friends may choose to send them.

"'8. And in order to prevent ambiguity in the interpretation of this act, be it understood, that herein the word 'physician' implies any fellow, candidate or licentiate of the colleges of physicians in the United Kingdom; that the word 'surgeon' implies any member of the colleges of surgeons; and the term 'apothecary' any licentiate or member of the companies of apothecaries, in the aforesaid kingdom.

"'9. And be it enacted, that the following shall be the form of the certificate, signed by a physician, surgeon, or apothecary, referred to above:—

"'I testify that I have opened the of the body of and having found therein am of opinion was the cause of his death.

"'(Signed)'"

"'10. And be in enacted, that any person interring, or aiding and abetting of the interment of a body, the said person or persons knowing the same to be unopened; also, all surgeons refusing to perform, or neglecting the fourth and fifth clauses of this act, shall be committed to the county gaol, for any term not exceeding one calendar month, by any two justices of the peace."

Such an impracticable, disgusting, and utterly useless bill was never engendered in the brain of man. Thus anatomy is to be learnt—not by the pupil—but after he has become a physician, surgeon, or apothecary—and that by a hasty *post mortem* examination in the presence of idle or curious gossips! This levelling of all distinctions, this wanton laceration of all feeling, this cutting up, *en masse*, this needless and profligate waste of subjects, is all for the purpose of familiarising the minds of the public with anatomy! How any man, in his senses, could propose such general and indiscriminate slaughtering, after exhibiting such acute sensibility towards the outrage committed on the spirit of the British constitution by the private dissection of unclaimed bodies, it is difficult to imagine. The whole pamphlet, indeed, would lead us to suspect that it is written by some enemy to anatomy, who wishes, in this underhand manner, to harrow up the most turbulent passions of the community, and raise still higher the public prejudice against the dissection of human bodies!

---

